# Supplementary material for: WordCommentsAnalyzer: A windows software tool for qualitative research
Source: F1000Res. 2018 Sep 4;7:536. Originally published 2018 May 3. [Version 2] doi: 10.12688/f1000research.14819.2 (PMC6137411; doi:10.12688/f1000research.14819.2)
Supplement: Supplementary file 3 [file f1000research-7-17614-s0002.tgz › b2a79496-4ada-47c4-9682-a99ce867836d.zip!/Eight Iranian Health Journals/Journal of Research in Rehabilitation Sciences _35.docx]

© Journal of Research in Rehabilitation Sciences . This work is licensed under CC BY-NC 4.0 (https://creativecommons.org/licenses/by-nc/4.0/).

Volume & Issue: دوره 8، شماره 6: 1391

Title: بررسی رابطه بین وضعیت جلو آمدن سر و نقاط ماشه‌ای در عضله تراپزیوس با گردن‌درد مزمن

http://jrrs.mui.ac.ir/index.php/jrrs/article/view/583

مقدمه: گردن‌درد مزمن یکی از علل شایع مراجعه افراد به مراکز فیزیوتراپی است که ممکن است در ارتباط با وضعیت نامناسب بدنی از جمله قرار گرفتن طولانی سر، گردن و شانه‌ها در وضعیت نامناسب در حین کارهای روزمره باشد. این تحقیق به منظور بررسی رابطه گردن درد مزمن و نقاط ماشه‌ای با جلو آمدن سر در افراد مبتلا به گردن‌درد در محدوده سنی 60-20 سال در مقایسه با افراد سالم انجام شد. مواد و روش‌ها: در پژوهش حاضر، 31 بیمار دارای درد مزمن گردن و 31 فرد سالم شرکت داشتند. توسط یک دوربین دیجیتال از افراد و از نمای جانبی راست عکس‌هایی تهیه شد و با استفاده از نرم‌افزار ImageJ زوایای جلو آمدن سر و شانه اندازه‌گیری شد. از آزمون‌های t و 2c برای تحلیل اطلاعات استفاده شد. یافته‌ها: ارتباط معنی‌داری بین درد مزمن گردن و جلو آمدن سر به دست آمد (0001/0 > P). به این صورت که در افراد بیمار نسبت به افراد سالم میانگین زوایای جلو آمدن سر کمتر بود، یعنی جلوآمدگی سر بیشتر بود. همچنین ارتباط معنی‌داری بین درد مزمن گردن و جلو آمدن شانه وجود داشت (0001/0 > P). به این صورت که در افراد بیمار نسبت به افراد سالم جلوآمدگی شانه بیشتر بود. ارتباط قوی و معنی‌داری بین گردن‌درد مزمن با وجود نقاط ماشه‌ای در عضله تراپزیوس دیده شد (0001/0 > P). نتیجه‌گیری: با توجه به وجود رابطه بین گردن درد مزمن و جلو آمدن سر و شانه می‌توان در برنامه درمانی این بیماران بر اصلاح وضعیت‌های نامناسب سر و شانه تأکید کرد. همچنین با توجه به رابطه نقاط ماشه‌ای عضله تراپزیوس با گردن‌درد پیشنهاد می‌گردد که در معاینه بیماران مبتلا به گردن‌درد مزمن وجود این نقاط ماشه‌ای بررسی شود و در درمان این بیماران به کمک روش‌های مختلف فیزیوتراپی این نقاط خاموش گردند.

Title: مقایسه نتایج آزمون فاصله در نویز در افراد دارای شنوایی هنجار با مبتلایان به وزوز گوش

http://jrrs.mui.ac.ir/index.php/jrrs/article/view/646

مقدمه: آزمون فاصله در نویز (Gaps in noise یا GIN)، مهارت وضوح زمانی شنوایی را ارزیابی می‌نماید. مطالعات قبلی نشان داده‌اند که آزمون فاصله در نویز، آزمونی با کاربردی آسان است که دارای حساسیت و ویژگی خوبی است. هدف مطالعه حاضر، ارزیابی مهارت وضوح زمانی شنوایی در افراد دارای شنوایی هنجار با و بدون وزوز بود. مواد و روش‌ها: این مطالعه هم‌گروهی همزمان غیر مداخله‌ای روی 20 فرد دارای وزوز (14 نفر وزوز دو طرفه و 6 نفر دارای وزوز یک طرفه) با آستانه شنوایی هنجار، با میانگین سنی 3/30 سال به عنوان گروه بیمار و 20 فرد با شنوایی هنجار بدون وزوز با میانگین سنی 8/27 به عنران گروه شاهد با شرایط سنی و جنسیتی مشابه در دانشکده توان‌بخشی دانشگاه علوم پزشکی تهران انجام گرفت. پس از انجام آزمون فاصله در نویز GIN، آستانه تقریبی و درصد پاسخ‌های صحیح افراد استخراج شد و با آزمون ناپارامتری  Mann-Whitneyمورد تجزیه و تحلیل قرار گرفت. یافته‌ها: آستانه تقریبی در گوش راست و گوش چپ گروه بیمار به طور معنی‌داری طولانی‌تر از گروه شاهد بود (به ترتیب 004/0 = P و 001/0 = P). درصد پاسخ‌های صحیح در گوش راست و گوش چپ گروه بیمار به طور معنی‌داری کمتر از گروه شاهد بود (به ترتیب 007/0 = P و 001/0 = P). نتیجه‌گیری: نتایج آزمون فاصله در نویز، نقص در پردازش وضوح زمانی شنوایی افراد دارای وزوز با شنوایی هنجار را نشان داد. بنابراین می‌توان این طور برداشت کرد که عملکرد ضعیف‌تر پردازش شنوایی در افراد دچار وزوز محتمل خواهد بود.

Title: بررسی تأثیر آموزش مهارت‌هاي زندگي بر مهارت‌هاي شناختی، رفتاری، هیجانی و انگیزشی دانش‌آموزان با اختلال رياضي

http://jrrs.mui.ac.ir/index.php/jrrs/article/view/589

مقدمه: مطالعات زیادی در رابطه با اثربخشی آموزش مهارت‌های زندگی بر هر یک از مهارت‌های شناختی، رفتاری، هیجانی و انگیزشی دانش‌آموزان عادی انجام شده است؛ در حالی که مطالعات اندکی به بررسی تأثیر آموزش مهارت‌های زندگی بر تمام متغیرهای بالا به طور همزمان در دانش‌آموزان با اختلال رياضي پرداخته‌اند. هدف این پژوهش، بررسی اثربخشي مهارت‌هاي زندگي بر مهارت‌های شناختی، رفتاری، هیجانی و انگیزشی دانش‌آموزان با اختلال رياضي بود. مواد و روش‌ها: پژوهش حاضر، یک مطالعه نیمه تجربی با طرح پیش‌آزمون- پس‌آزمون و گروه شاهد بود. 40 دانش‌آموز پسر 13 تا 15 ساله با  اختلال رياضي که به روش تصادفي از مدارس اختلال يادگيري شهرستان‌های تهران انتخاب شده بودند، در این پژوهش شرکت داشتند. شرکت کنندگان در گروه آزمایشی، آموزش مهارت‌های زندگی را در 9 جلسه دریافت کردند؛ در حالی که به گروه شاهد این آموزش ارایه نشد. این دانش‌آموزان به صورت تصادفی به دو گروه 20 نفری تقسیم شدند (يك گروه آزمایش و یک گروه شاهد). ابزارهاي استفاده شده در اين پژوهش آزمون هوشی Wechsler، آزمون تشخیصی رياضي Comet، برنامه آموزش مهارت‌های زندگی سازمان بهداشت جهاني و پرسش‌نامه خودتوانمندسازي اجتماعی Fleener، Lisa و Phillips بود. اطلاعات جمع‌آوری شده با استفاده از تحلیل واریانس چند متغيري در نرم‌افزار SPSS تحلیل شد. یافته‌ها: نتایج کلی تحلیل واریانس چند متغیری روی میانگین تفاوت نمره‌های پیش‌آزمون و پس‌آزمون نشان داد که گروه آزمایش و شاهد حداقل در یکی از 4 خرده مقیاس مهارت‌ شناختی، رفتاری، هیجانی یا انگیزشی تفاوت معنی‌داری دارند (27/13 = F و 001/0 > P). نتاج نهایی تحلیل واریانس چند متغیری نشان داد که آموزش مهارت‌های زندگی بر مهارت‌ شناختي (78/8 = F و 01/0 > P)، مهارت‌ رفتاري (37/22 = F و 05/0 > P)، کفایت‌ هيجاني (51/5 = F و 01/0 > P) و آمایه‌ انگيزشي (42/17 = F و 01/0 > P) دانش‌آموزان با اختلال ریاضی اثر مثبت و معنی‌داری داشت. نتیجه‌گیری: آموزش مهارت‌های زندگی بر مهارت‌های‌ شناختي،‌ رفتاري، هيجاني و‌ انگيزشي دانش‌آموزان با اختلال ریاضی اثر معنی‌داری داشت. با توجه به این که تقویت این مهارت‌ها منجر به خودتوانمندسازي اجتماعی دانش‌آموزان می‌شود، برنامه‌ریزی جهت آموزش مهارت‌های زندگی به دانش‌آموزان با اختلال ریاضی اهمیت ویژه‌ای دارد.

Title: بررسی رابطه بین نقص عملکردهای بینایی و کیفیت زندگی نابینایان

http://jrrs.mui.ac.ir/index.php/jrrs/article/view/679

مقدمه: یکی از بارزترین گروه افراد دارای ناتوانی، معلولین کم‌بینا و نابینا هستند که وجود معلولیت و نقص بینایی وضعیت کیفیت زندگی آن‌ها را به شدت تحت تأثیر قرار می‌دهد. این مطالعه به ارزیابی رابطه نقص عملکردهای بینایی و کیفیت زندگی معلولین نابینا و کم‌بینا پرداخت. مواد و روش‌ها: مطالعه توصیفی- تحلیلی حاضر در سال 1391 و در شهر زاهدان انجام شد. روش نمونه‌گیری به صورت سرشماری بود و 121 نفر از معلولین نابینا و کم‌بینای عضو سازمان بهزیستی شهر زاهدان که بالای 7 سال سن داشته و معلولیت دیگری نداشتند، مورد بررسی قرار گرفتند. ابتدا معاینات بینایی شامل بررسی حدت بینایی، دید محیطی و دید عمق انجام شد. سپس اطلاعات با استفاده از دو پرسش‌نامه دموگرافیک و پرسش‌نامه کیفیت زندگی نابینایان (که در سال 1386 به فارسی ترجمه شده بود و در دانشگاه علوم پزشکی اصفهان تدوین و پایایی آن به روش آزمون مجدد برآورد و مقدار همبستگی آن 89/0 به دست آمده بود) جمع‌آوری شد. در نهایت داده‌ها با استفاده از نرم‌افزار  SPSS نسخه 20 تجزیه و تحلیل گردید. یافته‌ها: میانگین کیفیت زندگی در زنان پایین‌تر از مردان بود. نتایج آزمون t این اختلاف را معنی‌دار نشان داد (001/0 > P). نتایج آزمون Mann-Whitney U نشان داد که افراد بدون دید عمق در بعد فراغت (009/0 = P) و اجتماعی (003/0 = P)، و افراد دارای دید تونلی در بعد خودمراقبتی و تحرک (001/0 > P) به طور معنی‌داری نسبت به افراد طبیعی نمره کیفیت زندگی پایین‌تری داشتند. نتیجه‌گیری: نقص دید محیطی، دید عمق، جنسیت و سطح تحصیلات اثر قابل توجهی بر کیفیت زندگی نابینایان در این منطقه دارد. با توجه به نتایج پژوهش، لزوم آموزش و ارایه برنامه‌های اجتماعی و تفریحی برای معلولین به خصوص زنان و همچنین انجام پژوهش‌های بیشتر بسیار ضروری است.

Title: طراحی و ساخت یک سیستم تعلیق مکشی سوکت جدید برای پروتز زیبایی سیلیکونی انگشت

http://jrrs.mui.ac.ir/index.php/jrrs/article/view/588

مقدمه: آمپوتاسیون‌های انگشت یا بخشی از انگشت،رایج‌ترین شکل قطع عضو می‌باشد. قطع انگشتان نه تنها مسایل روان‌شناختی و اجتماعی فرد را تحت تأثیر قرار می‌دهد، بلکه می‌تواند روی مسایل اقتصادی فرد و خانواده‌اش نیز تأثیر منفی گذارد. و استفاده از پروتز نیز روی مسایل اجتماعی، روان‌شناختی و اقتصادی فرد و خانواده‌اش تاثیرگذاراست. زیبایی ظاهری و تعلیق مطمئن دو عامل مهم در عملکرد و استفاده از پروتز انگشت می‌باشند. هدف از این مطالعه، طراحی و اصلاح یک سیستم ساکشن سوکت جدید برای پروتزهای زیبایی سیلیکونی انگشت بود. مواد و روش‌ها: در مطالعه حاضر پروتز زیبایی سیلیکونی شیاردار انگشت و پروتز زیبایی سیلیکونی معمولی انگشت برای 30 نفر آمپوته (7 زن و 23 مرد، میانگین سنی 4/29 سال) انگشت طراحی و ساخته شد و قدرت تعلیق در هر دو نوع سیستم تعلیق با استفاده از نیروسنج با دقت 01/0نیوتن اندازه‌گیری شد. یافته‌ها:حداکثر نیروی تعلیق ساکشن سوکت در پروتز جدید و پروتز معمولی به ترتیب 27/1 و 8/0 نیوتن می‌باشد (001/0 > P) که بیانگر عملکرد بهتر سیستم جدید نسبت به سیستم قبلی می‌باشد و می‌توان این برتری را ناشی از ایجاد حلقه‌هایی در سطح داخلی سوکت سیلیکونی دانست. نتیجه‌گیری: طراحی و ساخت پروتز سیلیکونی انگشت با روش جدید می‌تواند علاوه بر ایجاد تعلیق مطمئن‌تر نسبت به سیستم قبلی، مانع از صرف هزینه مجدد برای ساخت پروتز پس از چند ماه استفاده از پروتز گردد.

Title: مقايسه کودکان داراي اختلال کمبود توجه- بيش‌فعالي و عادي در دو مقوله هوش هيجاني و سبک‌هاي دلبستگي

http://jrrs.mui.ac.ir/index.php/jrrs/article/view/597

مقدمه: مشکلات و ناسازگاري‌هاي‌ کودکان مبتلا به اختلال کمبود توجه- بيش‌فعالي لزوم توجه بيشتر به اين اختلال را روشن‌تر مي‌سازد. هدف پژوهش حاضر، مقايسه هوش هيجاني و سبک دلبستگي در کودکان مبتلا به اختلال کمبود توجه- بيش‌فعالي با کودکان عادي بود. مواد و روش‌ها: براي دستيابي به اين هدف، از بين مدارس راهنمايي شهر کرمانشاه در سال تحصيلي 90-1389 تعداد 11 مدرسه (1124 دانش‌آموز) به صورت تصادفي انتخاب و پس از شناسايي دانش‌آموزان مبتلا به اختلال کمبود توجه- بيش‌فعالي (تعداد = 120 نفر)، تعداد60 دانش‌آموز داراي ADHD (Attention deficit-hyperactivity disorder) با روش نمونه‌گيري تصادفي ساده انتخاب شدند. سپس، پرسش‌نامه‌هاي‌ هوش هيجاني شوت (SEIS یا Schutte emotional intelligence scale) و پرسش‌نامه پيوند والديني (Parental bonding instrument یا PBI) Parker و همکاران را تکميل کردند، همچنين روش پژوهش حاضر، تحليلي از نوع علي- مقايسه‌اي (مقطعی) بوده و براي تحليل داده‌ها‌ از آزمون t استفاده شد. يافته‌ها‌: ميانگين زير مقياس‌هاي‌ هوش هيجاني در کودکان عادي به ترتيب، در تنظيم هيجان و ابراز هيجاني و در کودک مبتلا به اختلال کمبود توجه- بيش فعالي، ارزيابي هيجانات ديگران و ابراز هيجاني بيشتر از ساير مؤلفه‌ها‌ است. ميانگين نمره‌ دانش‌آموزان عادي در هوش هيجاني و مؤلفه‌هاي‌ آن از ميانگين نمره دانش‌آموزان مبتلا به اختلال کمبود توجه- بيش فعالی بيشتر است، به اين معني که در تمام اين مقوله‌ها‌، بين دانش‌آموزان عادي و کمبود توجه- بيش‌فعال تفاوت معني‌داري وجود دارد (01/0 > P). ميانگين نمره دانش‌آموزان عادي در مقوله‌هاي‌ مراقبت با محبت مادر و مراقبت با محبت پدر، از ميانگين نمره دانش‌آموزان مبتلا به کمبود توجه- بيش فعالي بيشتر بود (01/0 > P). همچنين، ميانگين نمره دانش‌آموزان مبتلا به کمبود توجه- بيش‌فعالي از ميانگين نمره‌ دانش‌آموزان عادي در مقوله‌هاي‌ کنترل مادر، کنترل پدر و سبک دلبستگي به طور کلي بيشتر مي‌باشد (01/0 > P). نتيجه‌گيري: بين كودكان مبتلا به اختلال كمبود توجه- بيش‌فعالي و كودكان عادي از نظر مؤلفه‌هاي‌ هوش هيجاني تفاوت‌هاي‌ معنی‌داري وجود دارد. كاربرد يافته‌هاي‌ اين پژوهش و محدوديت‌ها‌ و پيشنهادهای آن مورد بحث قرار گرفته است.

Title: مقایسه ساخت سازه‎ای واکه‌های بلند زبان فارسی در کودکان کاشت ‌حلزون ‎شده و کودکان دارای شنوایی طبیعی

http://jrrs.mui.ac.ir/index.php/jrrs/article/view/724

مقدمه: شنوایی یکی از عوامل اساسی تاثیرگذار بر کیفیت تولید گفتار از سوی سخنوران زبان است. این حس بازخورد لازم را برای کنترل گفتار فراهم می‎کند. گفتار کودکان دارای آسیب شنوایی اغلب هم از نظر ادراکی و هم از نظر ویژگی‎های آکوستیکی دارای اختلال می‎باشد. هدف از اين مطالعه، تعيين و مقايسه ساختار سازه‎اي سه واکه بلند زبان فارسی در کودکان کاشت ‌حلزون ‎شده و دارای شنوایی طبیعی بود. مواد و روش‎ها: صدای 20 کودک کاشت حلزون شده پیش ‎زبان و 20 کودک دارای شنوایی طبیعی در گروه سنی 5 تا 10 سال مورد بررسی قرار گرفت. شرکت‌کننده‎ها تک ‎زبانه و فارسی ‎زبان بودند. از آزمودنی‎ها خواسته شد که واکه‎های /i/‎، /u/ و ‎/â/‎‎ را کشیده و تولید کنند. میانگین سازه‎های اول و دوم این واکه‎ها با نرم‎افزار  Praatنسخه 5.3.13 تعیین و یافته‎ها بین دو گروه مقایسه گردید. جهت تعيين وجود تفاوت بين ميانگين سازه‎های اول، دوم و نسبت سازه دوم به اول بین دو گروه از آزمون Independent t استفاده شد. یافته‎ها: مقدار سازه اول هر سه واکه در گروه کاشت افزایش یافت که این افزایش تنها در واکه /i/ معنی‌دار بود (011/0 =P ). مقایسه‎ سازه‎ دوم تفاوت معنی‌داری را بین دو گروه نشان نداد. مقایسه نسبت سازه دوم به اول در دو گروه در سه واکه تنها در واکه /i/ و با احتمال 95 درصد تفاوت معنی‌دار نشان داد (01/0 =P ). نتیجه‎گیری: بسامد سازه‎های اول در کودکان کاشت ‌حلزون ‎شده افزایش یافت. با توجه به این یافته یعنی افزایش سازه اول، به نظر می‎رسد کودکان کاشت‌ حلزون ‎شده حرکات جبرانی زیادی به کار می‎گیرند که می‎توان این پدیده را با فقدان بازخورد شنیداری و تلاش برای جبران آن با حس عمقی با استفاده از شگردهای تولیدی توضیح داده شود. تغییر سازه‎های اول و دوم در جهت کاهش فضای هندسی واکه‎های مورد بررسی بود، به عبارتی افرادی که کاشت حلزون ‎شده بودند اندکی مرکزی شده‎تر واکه‎ها را تولید نمودند.

Title: بررسی تکرارپذیری استفاده از سونوگرافی برای ارزیابی ضخامت عضلات دیواره شکم در زنان سالم و مبتلا به کمردرد مزمن غیر اختصاصی

http://jrrs.mui.ac.ir/index.php/jrrs/article/view/775

مقدمه: کمردرد از شایع‌ترین اختلالات سیستم عضلانی- اسکلتی می‌باشد که به طور تقریبی 80 درصد افراد در طول زندگی خود حداقل یک بار آن را تجربه کرده‌اند. با توجه به نقش عضلات شکم در ثبات کمری- لگنی، بررسی ضخامت و عملکرد این عضلات نیازمند ابزاری با تکرارپذیری بالا است. هدف از انجام این مطالعه، تعیین تکرارپذیری سونوگرافی در ارزیابی ضخامت عضلات دیواره شکم در زنان سالم و مبتلا به کمردرد مزمن غیر اختصاصی بود. مواد و روش‌ها: مطالعه متدولوژیک حاضر روی 10 نفر، 5 زن سالم و 5 زن مبتلا به کمردرد مزمن غیر اختصاصی انجام گرفت. ضخامت عضلات دیواره شکم با استفاده از دستگاه سونوگرافی و اندازه‌گیری‌ها دو بار در یک روز با فاصله زمانی 2 ساعت و بار سوم با فاصله 5 روز از اندازه‌گیری روز اول انجام و ثبت شدند. آزمون ضریب همبستگی (Intraclass correlation coefficient یا ICC) برای تکرارپذیری نسبی و آزمون خطای معیار اندازه‌گیری برای بیان تکرارپذیری مطلق استفاده گردید. یافته‌ها: یافته‌های حاصل برای آزمون تکرارپذیری نسبی در همه تست‌ها به جز عضله مایل داخلی راست و عضله عرضی سمت چپ عالی بود و در افراد مبتلا به کمردرد در دامنه متوسط تا خوب بود. همه تست‌ها دارای تکرارپذیری مطلق بودند. نتیجه‌گیری: روش سونوگرافی به عنوان یک روش تکرارپذیر جهت بررسی ضخامت عضلات دیواره شکم می‌تواند استفاده شود.

Title: شواهد عصب شناختی نقصان برتری طرفی مغز در کودکان مبتلا به اختلال طیف اتیسم

http://jrrs.mui.ac.ir/index.php/jrrs/article/view/582

مقدمه: منحصر به فردترین جنبه در سازمان‌بندی مغز انسان عدم تقارن مغزی است و اوتیسم یکی از اختلالاتی است که شاید بر این عدم تقارن و عملکرد نیمکره‌های مغزی تأثیر می‌گذارد. هدف از این مطالعه، بررسی تفاوت عدم تقارن نیمکره‌ای در افراد اوتیسم نسبت به همتایان عادی و تعیین ارتباطات بین طرفی شدن دست و زبان بود. مواد و روش‌ها: در مطالعه حاضر 26 پسر مبتلا به اوتیسم با عملکرد بالا، با استفاده از ASSQ و 27 پسر همتای عادی (سن و هوش‌بهر) از دبستان‌های پسرانه شهرستان نجف آباد انتخاب شدند. تمام نمونه‌ها با استفاده از پرسش‌نامه برتری طرفی Edinburgh، آزمون ضربه زدن و آزمون شنود دو گوشی مورد ارزیابی قرار گرفتند. پس از جمع‌آوری داده‌ها، تحلیل با استفاده از نرم‌افزار SPSS نسخه 19 انجام شد. یافته‌ها : 2/19 درصد نمونه‌های اوتیستیک برتری دست چپ داشتند و تقاوت معنی‌داری بین نمرات Edinburgh دستی دو گروه وجود دارد (006/0 = P). بین دو دست افراد اوتیستیک در آزمون ضربه زدن تفاوتی مشاهده نشد (186/0 = P) و تفاوت دو دست در دو گروه معنی‌دار بود (021/0 = P). در آزمون شنود دو گوشی مزیت گوش راست در افراد اوتیستیک مشاهده شد (001/0 > P)، ولی تفاوت معنی‌داری بین دو گوش در دو گروه نیز مشخص شد (007/0 = P). نتایج هیچ گونه همبستگی بین عوامل برتری دستی و طرفی شدن زبان آشکار نکرد. نتیجه‌گیری : طرفی شدن طبیعی در هیچ یک از آزمون‌های ضربه زدن و شنود دو گوشی و پرسش‌نامه برتری دستی Edinburgh در افراد اوتیستیک مشاهده نشد و عدم تقارن نیمکره‌ای طبیعی در افراد اوتیستیک از الگوی هنجاری پیروی نمی‌کند. شیوع بالای چپ دستی در افراد اوتیستیک نیز عاملی بر تأیید ناهنجاری در عملکرد بین نیمکره‌ای می‌باشد. به علاوه با وجود ارتباط نزدیک برتری دستی و طرفی شدن زبان، هیچ همبستگی مشخصی بین این عوامل مشاهده نشد.

Title: بررسي تأثير نوع بار (ديناميك يا استاتيك) بر فعاليت عضلات تنه

http://jrrs.mui.ac.ir/index.php/jrrs/article/view/680

مقدمه: کمردرد ناشی از فعاليت‌های باربرداری و نگهداشتن بار، عامل مهمی در ايجاد ناتوانی عملکردی برای بيمار و ضررهای سنگين اقتصادی است. هنگام نگهداری بارهای ديناميکي مقدار گشتاور وارد شده به ستون فقرات در حال تغيير است که می‌تواند ثبات ستون فقرات را به مخاطره بيندازد. هدف از اين تحقيق، بررسی تأثير نوع بار (ديناميك يا استاتيك) بر فعاليت عضلات تنه به منظور بررسي دقيق‌تر ضايعات ستون فقرات بود. مواد و روش‌ها: 12 مرد سالم در اين مطالعه شرکت کردند. افراد بارهای ديناميک و استاتيك را در دو وضعيت ايستاده و خميده تنه (12 آزمون)، در یک جلسه آزمايش حفظ کردند. فعاليت عضلات [درصد ماكزيمم انقباض ارادي (Maximum voluntary contraction یا MVC)] راست شكمي، مايل داخلي و خارجي شكمي، اركتوراسپاين پشتي و كمري و مولتي فيدوس مورد ارزيابي قرار گرفت. يافته‌ها: فعاليت عضله اركتوراسپاين كمري حين نگهداري بارهاي ديناميكي در مقايسه با بار استاتيك به طور معني‌داري افزايش يافت. فعاليت عضلات شكمي، مولتي فيدوس و اركتوراسپاين پشتي حين نگهداري بارهاي ديناميكي افزايش يافت، اما معني‌دار نبود. نتيجه‌گيری: افزايش فعاليت عضلات اركتوراسپاين حين حفظ بارهاي ديناميكي منجر به افزايش نيروهاي فشاري روي ستون فقرات مي‌گردد. بنابراين جهت جلوگيري از بروز آسيب، علاوه بر مقدار بار (كيلوگرم)، نوع بار خارجي (استاتيك يا ديناميك) نيز بايد در فعاليت‌های باربرداری و نگهداشتن بار مورد توجه قرار گيرد.

Title: شیوع اختلالات شنوایی در کودکان 3 تا 6 ساله مهد کودک‌های شهر همدان

http://jrrs.mui.ac.ir/index.php/jrrs/article/view/684

مقدمه: هر گونه نقص قابل توجه در عملکرد حس شنوایی، مشکلاتی را در برقراری ارتباط و سازگاری با محیط ایجاد می‌کند. هدف از انجام این مطالعه، تعیین شیوع اختلالات شنوایی در کودکان سنین 3 تا 6 ساله مهد کودک‌های شهر همدان در سال 1391 بود. مواد و روش‌ها: مطالعه‌ی حاضر از نوع توصیفی- تحلیلی و به روش مقطعی بر روی 345 کودک (185 دختر و 160 پسر) انجام گرفت. نمونه‌گیری به صورت تصادفی ساده بود. پس از تکمیل برگه آگاهی از وجود کم‌شنوایی توسط والدین و مربیان، معاینه اتوسکوپی، آزمون Immittance و غربالگری تون خالص صورت گرفت. یافته‌ها: 6/11 درصد حالت‌های ناهنجار مجرای گوش، 6/22 درصد اختلال پرده تمپان، 8/22 درصد تمپانوگرام ناهنجار، 7/28 درصد عدم وجود رفلکس صوتی و 9/11 درصد کم‌شنوایی مشاهده شد. کم‌شنوایی در 3/10 درصد موارد از نوع انتقالی، 3/1 درصد از نوع حسی عصبی و در 3/0 درصد از نوع آمیخته بود. در 9/11 درصد از کودکان دارای کم‌شنوایی، کم‌شنوایی خفیف تا متوسط مشاهده گردید و هیچ موردی از کم‌شنوایی شدید و عمیق مشاهده نشد. 96 نفر از کودکان (8/27 درصد) نیازمند اقدامات درمانی و 6 نفر (7/1 درصد) نیازمند اقدامات توان‌بخشی بودند. میزان آگاهی والدین (3/2 درصد) و مربیان (6/1 درصد) از وجود کم‌شنوایی در سطح پایینی قرار داشت. نتیجه‌گیری: نظر به وجود درصد بالایی از کودکان نیازمند به اقدامات درمانی، تأثیر اختلالات شنوایی بر یادگیری کودکان در سنین پایین و اهمیت تشخیص و مداخله زود هنگام در موارد وجود کم‌شنوایی، اجرای برنامه‌های توان‌بخشی شنوایی، آگاه نمودن مسؤولین، مربیان مهد کودک‌ها و والدین کودکان و انجام دوره‌ای برنامه غربالگری شنوایی در سنین پایین پیشنهاد می‌شود.

Title: بررسی تاثیر یک دوره تمرین درآب بر افسردگی مردان سالمند

http://jrrs.mui.ac.ir/index.php/jrrs/article/view/629

مقدمه: افسردگی یکی از شایع‌ترین اختلالات روانی و معضلات دوره سالمندی است. هدف از پژوهش حاضر، بررسی اثر یک دوره تمرین درمانی در آب بر میزان افسردگی مردان سالمند بود. مواد و روش‌ها: 30 مرد سالمند (سن 19/5 ± 46/68 سال، وزن 72/4 ± 66/64 کیلوگرم، قد 25/4 ± 6/167 سانتی‌متر) به طور تصادفی انتخاب و در دو گروه مورد و شاهد تقسیم شدند. گروه مورد به مدت 6 هفته (سه جلسه در هفته) تمرین درمانی در آب را اجرا کرده و گروه شاهد فعالیت معمول روزانه خود را اجرا نمودند. میزان افسردگی آزمودنی‌های پژوهش در دو مرحله قبل و بعد از اجرای دوره تمرین درمانی در آب با کمک پرسش‌نامه افسردگی Beck تحت بررسی قرار گرفت. تحلیل داده‌ها با استفاده از آزمون‌های‌ Independent t و Paired t در سطح معنی‌داری (05/0 £ P) اجرا گردید. یافته‌ها: بین دو گروه مورد و شاهد در پیش‌آزمون در میزان نمرات افسردگی تفاوت معنی‌داری وجود نداشت، اما در پس‌آزمون تفاوت معنی‌داری دیده شد. نتایج آزمون Paired t نیز اختلاف معنی‌دار نمرات افسردگی بین پیش و پس‌آزمون را در گروه مورد نشان داد؛ در حالی که بین پیش و پس‌آزمون گروه شاهد تفاوت معنی‌داری مشاهده نگردید. نتیجه‌گیری: با توجه به نتایج تحقیق حاضر، چنین به نظر می‌رسد که تمرین درمانی در آب بر کاهش شدت افسردگی مردان سالمند مؤثر است و می‌تواند به عنوان یک روش مداخله‌ای تأثیرگذار مورد توجه قرار گیرد.

Title: بررسی تأثیر استفاده از وسایل کمکی بر کیفیت زندگی مادران کودکان مبتلا به فلج مغزی

http://jrrs.mui.ac.ir/index.php/jrrs/article/view/867

مقدمه: نقایص کارکردی در کودکان فلج مغزی منجر به محدودیت مشارکت آنان در انجام فعالیت‌های روزمره زندگی و در نتیجه اعمال فشارهای جسمانی و روانی بر مراقبین می‌گردد که این مسأله می‌تواند تأثیر منفی بر کیفیت زندگی مادران داشته باشد. پژوهش حاضر با هدف، بررسی تأثیر وسایل کمکی بر کیفیت زندگی مادران کودکان فلج مغزی انجام شد. مواد و روش‌ها: در مطالعه نیمه تجربی حاضر، 291 نفر از مادران دارای کودک فلج مغزی 0 تا 14ساله ساکن شهر تهران، به شیوه نمونه‌گیری در دسترس انتخاب شدند. سپس با توجه به سطوح عملکرد حرکتی درشت و توانایی دستی کودک فلج مغزی، وسایل کمکی در اختیار کودکان قرار گرفت. کیفیت زندگی مادران با استفاده از پرسش‌نامه کیفیت زندگی 36-SF (36 Short form) شدت فلج مغزی با سیستم طبقه‌بندی عملکرد حرکتی درشت (Gross motor function classification system یا GMFCS) و توانایی دستی با سیستم طبقه‌بندی توانایی دستی مورد بررسی قرار گرفت. اطلاعات جمع‌آوری شده با شاخص‌های مرکزی و پراکندگی و با استفاده از آزمون آماری Paired t تحلیل گردید. یافته‌ها: میانگین سنی مادران در این گروه، 69/36 سال و انحراف معیار 15/6 بود. نتایج نشان دهنده افزایش معنی‌داری در میانگین نمرات محدودیت ایفای نقش به دلایل مشکلات فیزیکی (001/0 = P)، درد جسمانی (004/0 = P)، سلامت عاطفی (001/0 > P) و نشاط (001/0 > P) و همچنین در ابعاد کلی سلامت روانی (008/0 = P)، سلامت جسمانی (006/0 = P) و کیفیت کلی زندگی (001/0 = P) قبل و بعد از مداخله بود، ولی در سایر خرده مقیاس‌ها اختلاف معنی‌داری مشاهده نگردید (05/0 < P). نتیجه‌گیری: با توجه به نتایج فوق، وسایل کمکی بر ارتقای کیفیت زندگی مادران کودکان فلج مغزی مؤثر است.

Title: تاثـير برنامه پيـشگيري کننده آسيب فيفا +11 بر قدرت ايزومـتريک عضلات چهارسر و همسترينگ فوتباليست هاي جوان حرفه اي ايران

http://jrrs.mui.ac.ir/index.php/jrrs/article/view/720

مقدمه: مفصل زانو یکی از شایع‌ترین نقاط بروز آسیب در فوتبالیست‌ها است که قدرت عضلانی به عنوان یکی از مهم‌ترین عوامل پیشگیری از آسیب‌های عضلانی و رباطی می‌باشد. هدف تحقيق حاضر، بررسي تأثير برنامه پيشگيري کننده آسيب فيفا +11 بر قدرت ايزومتريک عضلات همسترينگ و چهار سر ران فوتباليست‌هاي مرد جوان حرفه‌اي ايران بود. مواد و روش‌ها: 24 فوتباليست حرفه‌اي در قالب دو گروه تجربي و شاهد به صورت هدفمند انتخاب و گروه تجربي، برنامه پيشگيري کننده آسيب فيفا +11 را به مدت 2 ماه، 3 جلسه در هفته در زمان عصر اجرا کردند. قدرت ايزومتريک عضلات هر دو گروه قبل و بعد از برنامه تمريني به وسيله دستگاه بايودکس مدل 3 در سه زاويه 30، 60 و 90 درجه مورد ارزيابي قرار گرفت. از آزمون‌هاي Paired t، Independent t و Two way repeated measure ANOVA براي تجزيه و تحليل داده‌ها استفاده گرديد. يافته‌ها: در گروه تجربي، قدرت ايزومتريک عضلات چهار ‌سر در زاويه 90 درجه آزمون نهايي به طور معني‌داري بيشتر از پيش آزمون می‌باشد (004/0 = P)؛ در حالي که در قدرت عضلات همسترينگ پيش‌آزمون و پس‌آزمون در هيچ کدام از زوايا تفاوت معنی‌داري مشاهده نشد (005/0 ³ P). بين دو گروه تجربي و شاهد، تفاوت معنی‌داري در حداکثر گشتاور عضلات چهار سر در زاويه 30 درجه (001/0 > P)، 60 درجه (01/0 = P) و 90 درجه (004/0 = P) مشاهده شد. در گروه تجربي در هر سه زاويه، تفاوت معنی‌داري در حداکثر گشتاور عضلات چهار سر (001/0 > P) و همسترينگ (001/0 > P) وجود داشت؛ به گونه‌اي که در عضلات چهارسر بيشترين مقدار حداکثر گشتاور در زاويه 90 درجه (6/303 نيوتن‌متر) و در عضلات همسترينگ در زاويه 30 درجه (1/155 نيوتن‌ متر) مشاهده شد. نتيجه‌گيري: به نظر مي‌رسد برنامه تمريني فيفا +11 بر افزايش قدرت عضلات چهار سر ران فوتباليست‌هاي حرفه‌اي جوان مؤثر بوده است. افزودن تمرينات بيشتر با هدف افزايش قدرت عضلات همسترينگ به برنامه تمريني فيفا +11 توصيه مي‌شود.

Title: رابطه بین حمایت روانی اجتماعی فرزندان با سلامت روان سالمندان

http://jrrs.mui.ac.ir/index.php/jrrs/article/view/643

مقدمه: پژوهش حاضر به منظور بررسی رابطه بین حمایت روانی- اجتماعی فرزندان با سلامت روان سالمندان انجام شد. مواد و روش‌ها: در مطالعه توصیفی حاضر، 100 نفر سالمند زن و مرد ساکن در سرای سالمندان و ساکن در منزل در شهر مشهد به شیوه نمونه‌گیری در دسترس انتخاب شدند و با استفاده از پرسش‌نامه سلامت عمومی (General health Questionnaire یا 28-GHQ) مورد آزمون قرار گرفتند. برای تجزیه و تحلیل داده‌ها از آزمون Independent t استفاده شد. یافته‌ها: بین میزان سلامت روانی سالمندان دارای حمایت و فاقد حمایت تفاوت معنی‌داری بود، بدین صورت که این میزان در سالمندان دارای حمایت روانی- اجتماعی بیشتر است (005/0 > P) و در زمینه تفاوت سلامت روانی بین زنان و مردان سالمند تفاوت معنی‌داری مشاهد نشد. نتیجه‌گیری: سالمندان فاقد حمایت‌های روانی- اجتماعی (آن‌هایی که در مراکز نگهداری سالمندان زندگی می‌کنند) نسبت به سالمندان دارای حمایت‌های روانی- اجتماعی (آن‌هایی که به همراه خانواده خود زندگی می‌کنند) از علایم آسیب‌شناسی روانی بیشتری رنج می‌برند.

Title: مقايسه قدرت عضلات تنه و پنجه دست دانش‌آموزان عادی و کم‌توان ذهني فعال و غير فعال

http://jrrs.mui.ac.ir/index.php/jrrs/article/view/707

مقدمه: قدرت عضلاني به دليل وضعيت ويژه افراد کم‌توان ذهني و نياز آن‌ها به داشتن زندگي مستقل اهميت بسياري دارد. تحقيقات مختلف اعلام کرده‌اند که زندگي بي‌تحرک يکي از علل ضعف جسماني و مشکلات سلامتي در اين افراد مي‌باشد. هدف از اين مطالعه، مقايسه قدرت عضلاني نوجوانان کم‌توان ذهني و نوجوانان عادی فعال و غير فعال بود. مواد و روش‌ها: در اين تحقيق 60 دانش‌آموز 15 تا 20ساله (ميانگين سن13/1 ± 58/16سال) شامل 30 دانش‌آموز کم‌توان ذهني (ميانگين بهره هوشي 15/5 ± 87/62) و 30 دانش‌آموز عادي به روش تصادفی انتخاب شدند و به چهار گروه برابر کم‌توان ذهني فعال، کم‌توان ذهني غير فعال، عادی فعال و عادی غير فعال تقسيم شدند. قدرت عضلات پنجه دست برتر و تنه (پشت، سينه و شانه‌ها) با استفاده از نيروسنج‌هاي دستي و چندکاره ارزيابي شد. داده‌ها از طريق نرم‌افزار SPSS و آزمون MANOVA با آزمون تعقيبي LSD (Least significant difference) در سطح (05/0 £ P) تجزيه و تحليل شد. یافته‌ها: یافته‌های آزمون MANOVA نشان داد، در قدرت عضلاني پنجه دست برتر، تفاوت معنی‌داري بين گروه کم‌توان ذهني غير فعال با تمامی گروه‌هاي کم‌توان ذهني فعال (023/0 = P)، عادی غير فعال (016/0 =P) و عادی فعال (001/0 > P) وجود داشت. در قدرت عضلاني تنه نيز تفاوت معنی‌داري بين این گروه با گروه‌هاي کم‌توان ذهني فعال (008/0 = P)، عادی غير فعال (002/0 = P) و عادی فعال (001/0 > P) مشاهده شد؛ در حالی که بين گروه کم‌توان ذهني فعال با گروه عادی غير فعال در هر دو عامل قدرت پنجه دست (871/0 = P) و تنه (597/0 = P) تفاوت معنی‌دار نبود. البته بین نتایج این گروه با گروه عادی فعال در قدرت عضلانی تنه (016/0 = P)  تفاوت معنی‌دار بود، اما در قدرت عضلانی پنجه دست (064/0 = P) بین این دو گروه تفاوت معنی‌داری مشاهده نشد. همچنین در دانش‌آموزان عادی بین دو گروه فعال و غير فعال در هر دو عامل قدرت پنجه دست (090/0 = P) و قدرت تنه (056/0 = P) نیز تفاوت معنی‌داري مشاهده نشد. نتیجه‌گیری: نوجوانان کم‌توان ذهني با داشتن يک زندگي فعال مي‌توانند سطوح قدرت عضلاني را مانند همسالان عادی خود بهبود بخشند. به نظر مي‌رسد فعاليت‌هاي ورزشي آموزشگاهي مي‌تواند عامل مناسبي براي بهبود آمادگي عضلاني در افراد کم‌توان ذهني باشد.

Title: بررسی تأثیر جایگاه گوشی بر آستانه‌های ادیومتری صوت خالص در محدوده فرکانسی مرسوم

http://jrrs.mui.ac.ir/index.php/jrrs/article/view/358

مقدمه: بدون شک ادیومتری صوت خالص از پایاترین آزمون‌های موجود جهت بررسی مشکلات سیستم شنوایی است. جایگاه گوشی، از جمله عواملی است که تأثیر زیادی بر ارزیابی دقیق آستانه‌ها دارد. پژوهش حاضر با هدف، بررسی تأثیر جایگاه گوشی بر آستانهظهای ادیومتری صوت خالص در محدوده فرکانسی مرسوم انجام شد. مواد و روش‌ها: در مطالعه مقطعی حاضر، 50 فرد بزرگسال (25 زن و 25 مرد) 25-18 ساله به صورت تصادفی ساده، انتخاب و مورد ارزیابی قرار گرفتند. از تمام افراد تاریخچه‌گیری، اتوسکوپی و تمپانومتری گرفته شد و سپس آستانه‌های شنوایی آن‌ها در محدوده فرکانسی مرسوم (با گام‌های 5 و 1 دسی‌بل) در هر دو گوش تعیین گردید. گوشی مربوط، یک بار توسط فرد آزمایشگر و بار دیگر توسط فرد آزمایش شونده بر روی گوش‌ها قرار داده شد و آستانه‌های شنوایی افراد به روش نزولی کسب و ثبت گردیدند. جهت تجزیه و تحلیل نتایج از آزمون Paired t استفاده شد. یافته‌ها: تفاضل آستانه‌های حاصل از تغییر جایگاه گوشی در تمام فرکانس‌های مورد ارزیابی در هر دو گوش از نظر آماری معنی‌دار بود (05/0 > P). نتیجه‌گیری: جایگاه گوشی بر آستانه‌های شنوایی در فرکانس‌های مرسوم تأثیرگذار بود و به همین دلیل توصیه می‌گردد که در ارزیابی‌های بالینی، جایگذاری گوشی در هر شرایطی توسط شنوایی‌شناس مربوط صورت پذیرد.

Title: تأثیر برنامه کوتاه مدت راه رفتن مقاومتی روی تردمیل بر ارتباط پارامترهای ترکیب بدنی و سطوح هورمون‌های استرادیال، پروژسترون و کورتیزول در زنان یائسه استئوپروتیک

http://jrrs.mui.ac.ir/index.php/jrrs/article/view/622

مقدمه: با افزایش سن، سطوح هورمون‌های آنابولیک کاهش می‌یابد که زمینه‌ساز بسیاری از تغییراتی است که با افزایش سن در ترکیبات بدنی رخ می‌دهد. افزایش فعالیت بدنی می‌تواند در جبران کاهش هورمون در افراد مسن مورد توجه قرار گیرد. هدف از این مطالعه، بررسی تأثیر برنامه پیاده‌روی تحت بیشینه مقاومتی بر پارامترهای ترکیب بدنی و هورمون‌های استرادیال، پروژسترون و کورتیزول در زنان یائسه بود. مواد و روش‌ها: مطالعه حاضر از نوع کارآزمایی بالینی- تصادفی بود که روی 27 زن یائسه استئوپروتیک انجام شد. نمونه‌ها به صورت تصادفی در 3 گروه راه رفتن ساده روی تردمیل (8 نفر)، راه رفتن با جلیقه وزنه‌دار روی تردمیل (9 نفر) و شاهد (10 نفر) قرار گرفتند. پروتکل تمرین شامل 6 هفته برنامه راه رفتن تحت بیشینه با شدت 60-50 درصد ضربان قلب ذخیره، 3 بار در هفته و هر جلسه 30 دقیقه بود. در گروه جلیقه‌دار، افراد یک جلیقه وزنه‌دار (4 تا 8 درصد وزن بدن) می‌پوشیدند. متغیرهای ترکیب بدنی (نمایه توده بدن، توده چربی و توده غیر چربی) و سطوح هورمون‌های استرادیال، پروژسترون و کورتیزول قبل و بعد از 6 هفته اندازه‌گیری شد. یافته‌ها:کاهش توده چربی و افزایش توده غیر چربی در گروه جلیقه وزنه‌دار معنی‌دار بود (به ترتیب 027/0 = P و 005/0 = P). ارتباط بین توده چربی و توده غیر چربی در گروه‌های تمرینی معنی‌دار بود (به ترتیب 01/0 = P و 05/0 =P )؛ در حالی که بعد از 6 هفته سطح استرادیال و پروژسترون در سه گروه تغییر معنی‌داری نداشت. هورمون کورتیزول در گروه‌های تمرین کاهش معنی‌داری یافت، اما ارتباط معنی‌داری بین تغییرات سه هورمون به دست نیامد (05/0 < P). نتیجه‌گیری: در هر دو گروه تمرین با وجود این که ارتباط معنی‌دار بین متغیرهای ترکیب بدنی به دست آمد، اما اضافه کردن بار خارجی منجر به بهبودی بیشتری در سطح پارامترهای ترکیب بدنی گردید؛ در حالی که کورتیزول کاهش معنی‌داری نسبت به پایه در هر دو گروه تمرین داشت، اما تنها در گروه راه رفتن ساده روی تردمیل نسبت به کنترل معنی‌دار شد و به لحاظ سطح هورمون‌های آنابولیک اضافه کردن بار خارجی در مدت 6 هفته منجر به تغییرات معنی‌دار نشد.

Title: ارتباط تعادل ايستا و پويا با وقوع آسيب‌هاي اندام تحتاني در نوجوانان ورزشكار

http://jrrs.mui.ac.ir/index.php/jrrs/article/view/481

مقدمه: در بین نوجوانان، ورزش مهم‌ترین عامل وقوع حوادثی است که به مراقبت‌های پزشکی و اورژانس منجر می‌شود. هدف از انجام اين تحقيق، بررسي ارتباط تعادل ايستا و پويا با وقوع آسیب‌های اندام تحتانی در نوجوانان ورزشکار با استفاده از تست‌هاي عملكردي و باليني بود. مواد و روش‌ها: آزمودنی‌های این تحقیق100 نفر از پسران ورزشكار دبیرستان‌های شهرستان اهر در رشته‌هاي فوتبال، واليبال، بسكتبال و هندبال با ميانگين سني 44/1 ± 30/17سال، میانگین قد 43/7 ± 06/175سانتی‌متر و میانگین وزن 14/2 ± 92/69 کیلوگرم و با سابقه آسيب در انـــدام تحتاني در طي دو سال گذشته بودند که به صورت هدفمند انتخاب شدند. در این تحقیق از فـرم جمع‌آوري اطلاعات جهت ثبت آسيب‌هاي ورزشكاران، از آزمون تعادلي گردش ستاره براي سنجش تعادل پويا و از آزمون Stork براي ارزيابي تعادل ايستا استفاده شد. براي تجزيه و تحليل داده‌ها از روش‌های آمار توصيفي، ضريب همبستگی Pearson و آزمونFisher's Z  در سطح معني‌داري 05/0 تحت نرم‌افزار آماریSPSS  نسخه 17 استفاده شد. یافته‌ها: بين تعادل ايستا و پويا و وقوع آسیب‌هاي اندام تحتانی در پسران ورزشکار ارتباط منفي معني‌داری وجود داشت (05/0. > P، 64/0- = r). شاید ضعف و نقص در عضلات ثبات دهنده مرکزی و كنترل عصبي- عضلاني باعث تضعيف تعادل شده و نوجوانان ورزشکار را مستعد آسيب‌دیدگی مي‌كند. نتیجه‌گیری: با توجه به نتایج پژوهش به نظر می‌رسد که ضعف در تعادل ايستا و پويا، به عنوان یکی از عوامل اثرگذار بر آسیب‌دیدگی ورزشکاران نوجوان مورد توجه قرار گیرد. بنابراین، پیشنهاد می‌شود از تمرينات افزايش دهنده تعادل جهت كاهش وقوع آسيب‌ها در نوجوانان ورزشكار استفاده گردد.

Title: نقدی بر آموزش‌های هندلینگ (مراقبت ویژه از کودکان فلج مغزی): نامه‌ای به سردبیر

http://jrrs.mui.ac.ir/index.php/jrrs/article/view/705

خرسندیم که در مورد دو مقاله با عنوان «بررسی تأثیر هندلینگ (مراقبت‌های ویژه کودکان فلج مغزی) بر مراقبین و کودکان فلج مغزی» که در دوره 8، شماره 3: سال 1391 مجله پژوهش در علوم توان‌بخشی به چاپ رسیده‌اند چند نکته را مورد توجه قرار دهیم که به نظر می‌رسد توضیح آن برای خوانندگان مقالات ذکر شده ضروری باشد. 1- در مقالات ذکر شده تعاریف هندلینگ و نظر خود محققین در مورد هندلینگ عنوان نشده است. 2- جایگاه هندلینگ در کاردرمانی بیان نشده است. 3- این‌که هندلینگ در چه فعالیت‌هایی دیده می‌شود، مشخص نیست و 4- به تفاوت هندلینگ با فعالیت‌های روزمره زندگی نیز اشاره‌ای نگردیده است که به نظر می‌رسد ضرورت است به اختصار در مورد هر کدام از سؤالات فوق توضیحاتی داده شود.
